# Detection of bacterial DNA from central venous catheter removed from patients by next generation sequencing: a preliminary clinical study

**DOI:** 10.1186/s12941-018-0297-2

**Published:** 2018-12-22

**Authors:** Ken-ichi Okuda, Yutaka Yoshii, Satomi Yamada, Akio Chiba, Ippei Hironaka, Seiji Hori, Katsuhiko Yanaga, Yoshimitsu Mizunoe

**Affiliations:** 1 Department of Bacteriology, The Jikei University School of Medicine, 3-25-8 Nishi-Shimbashi, Minato-ku, Tokyo, 105-8461 Japan; 2 Jikei Center for Biofilm Science and Technology, The Jikei University School of Medicine, Tokyo, Japan; 3 Division of Respiratory Diseases, Department of Internal Medicine, The Jikei University School of Medicine, Tokyo, Japan; 4 Department of Surgery, The Jikei University School of Medicine, Tokyo, Japan; 5 Department of Infectious Disease and Control, The Jikei University School of Medicine, Tokyo, Japan

**Keywords:** Catheter-related infection, Diagnostics, Next-generation sequencing, 16S ribosomal DNA, Staphylococci

## Abstract

**Background:**

Catheter-related infection (CRI) is one of the serious challenges in clinical practice. This preliminary clinical study aimed to examine whether next-generation sequencing (NGS) targeting 16S rDNA, which was PCR-amplified directly from the tip of a central venous catheter (CVC), can be used to identify causative pathogens in CRI, compared to the culture method.

**Methods:**

Hospitalized patients, from whom a CVC had just been removed, were prospectively enrolled and divided into the CRI-suspected and routine removal groups. DNA was extracted from the sonication fluid of CVC specimens derived from patients. For analysis of bacterial composition by NGS, the V3–V4 fragments of bacterial 16S rDNA were PCR-amplified, followed by index PCR and paired-end sequencing on an Illumina MiSeq device. Conventional culture methods were also performed in the CRI-suspected group.

**Results:**

Of CVCs collected from the 156 enrolled patients (114 men; mean age 65.6 years), a total of 14 specimens [nine out of 31 patients suspected with CRI and five out of 125 patients without infection symptoms (routine removal group)] were PCR-positive. In five patients with definite CRI, *Staphylococcus* was the most frequently detected genus by NGS (4/5 specimens), although no pathogens were detected by NGS in the one remaining specimen. The genera identified by NGS were consistent with those from conventional culture tests. There was high agreement between NGS and the culture method in the CRI-suspected group, with sensitivity and specificity values of 80.0% and 76.9%, respectively; meanwhile, the false-positive rate of NGS was as low as 4.0% in the routine removal group. Moreover, several genera, besides the genus identified by culture test, were detected in each patient with definite CRI and surgical site infection (SSI). Additionally, in one patient with SSI, *Enterococcaceae* were detected not only by NGS but also by abdominal abscess drainage culture.

**Conclusions:**

NGS targeting 16S rDNA was able to analyze the bacterial composition of CVC specimens and detect causative pathogens in patients with CRI and was therefore suggested as a promising diagnostic tool for CRI.

## Background

Catheter-related infection (CRI), one of the major hospital-acquired infections, continues to be associated with morbidity, mortality, and additional medical cost [1–7]. As delayed start of the appropriate antibiotic treatment is a significant risk factor contributing to the worsening of CRI, accurate diagnosis of the causative pathogen is crucial for the survival of the patient. Although culture of blood and catheter samples is the standard process for a definitive diagnosis in clinical practice, catheter-related bloodstream infection frequently occurs despite negative results in blood culture, thereby delaying the relevant treatment [8, 9]. Moreover, if multiple pathogens are present in the specimen, the growth may become a bias in the conventional culture method, thereby rendering it impossible to obtain the true clinical picture. Hence, new diagnostic methods that can complement the limitations of culture testing to enable accurate detection of causative pathogens in CRI may be of clinical importance.

Polymerase chain reaction (PCR)-based methods for detecting causative pathogens in infectious diseases such as CRI have been widely used over the last 20 years since they are more rapid and sensitive than conventional culturing procedures [10, 11]. Nevertheless, there are some disadvantages of using PCR technology alone for detecting pathogens. For example, causative pathogens cannot be detected when specific primer sets are not included. Sanger sequencing of 16S rDNA, amplified using a universal primer set, can be applied when the specimen contains only one species, but it is usually impossible to simultaneously identify more than one species from a polymicrobial specimen without processing mixed chromatograms using a computer program [12]. However, CRI can be caused by various pathogens, such as *Staphylococcus*, *Enterococcus*, and non-fermenting Gram-negative rods, and sometimes appears as a mixed infection [13, 14]. Thus, development of new molecular diagnostic techniques is imperative to comprehensively detect multiple pathogens simultaneously from numerous potential candidates.

Recently, next-generation sequencing (NGS), which gives out millions or billions of sequence reads per instrument run, has attracted attention in molecular diagnosis of infections [15]. NGS enables culture-free and comprehensive detection of pathogens and acquirement of information about the microbiome in the specimens [16, 17]. Moreover, NGS can simultaneously identify all species in a polymicrobial specimen and is superior to Sanger sequencing in this regard [16]. Indeed, several studies have reported that single or multiple bacteria were identified as causative pathogens by NGS using blood [18, 19], urine [20], orthopedic [20], stool [21], pelvic [22], and abscess [23] samples from patients with sepsis, urinary tract infection, orthopedic infections, diarrhea, pelvic infection-induced inflammatory disease, and liver abscess, respectively. Although NGS of clinical samples can be a powerful diagnostic method for detecting causative pathogens, the usefulness of catheter samples from patients with CRI for NGS analysis has not been evaluated. Hence, we thought to apply NGS of catheter tip samples to more accurately detect the bacteria associated with CRI.

In this preliminary clinical study, we attempted to identify pathogens associated with CRI in patients by NGS targeting 16S rDNA. Unlike conventional culture methods, this method enables the identification of bacteria present on the catheter surface without a culture, as bacterial DNA is extracted directly from the catheter surface. Complementing these results with those from conventional methods, more reliable diagnosis may become possible. Moreover, in this study, we also sought to confirm whether this method could detect bacteria in patients with no infection symptom.

## Methods

### Patients and central venous catheter samples

This preliminary clinical study was prospectively conducted at the Jikei University Hospital from October 2012 to March 2014. Patients (aged 20 years or more), from whom a central venous catheter (CVC) had just been removed using sterile techniques, were eligible along with their CVCs for inclusion in the study. Other inclusion criteria were as follows: (1) the catheter had been inserted using maximal sterile barrier precautions, (2) in case the catheter was removed due to the suspicion of CRI, a culture of either a catheter tip specimen or blood specimen was considered for routine clinical examination. After written informed consent was obtained from each patient, the distal 60-mm segment of the removed catheter tip was cut and preserved in a freezer until the analysis of 16S rDNA PCR assay for targeted NGS was performed. If the catheter tip was already used for the conventional culture examination, the remaining was used for this analysis.

### Data collection

Data regarding age, sex, underlying disease, immunosuppressive drug use, body temperature, skin redness at the insertion site, CVC, blood and catheter tip cultures, and antibiotics were obtained from the patients’ medical records. Quick sequential (sepsis-related) organ failure assessment (qSOFA) score [24] was also obtained. Moreover, when the catheter was removed due to the suspicion of CRI, the definitive diagnosis was also recorded.

### Definition of the confirmed CRI

For the purpose of this study, CRI was originally defined as: (a) positive culture results of catheter tip or blood samples obtained from the peripheral vein or artery when CVC was removed, (b) clinical manifestations of infection (fever and skin redness) that improved following the removal of CVC, and (c) no apparent source of blood stream infection except for the catheter.

### Definition of the routine removal group

The patients who were judged as having no infection symptom by the physicians and whose catheter was removed because it was no longer needed were included in the routine removal group.

### Culture processing

In case CRI was suspected, blood and catheter tip samples were collected and processed at the clinical laboratory of our hospital. For blood culture, after inoculation of each blood sample in aerobic and anaerobic blood culture bottles (Becton–Dickinson, Sparks, MD, USA), they were incubated in an automated continuous monitoring blood culture system, BACTEC FX (Becton–Dickinson), for a maximum of 5 days. If bacteria had grown, Gram staining of the blood culture fluid was performed. Moreover, if the aerobic culture was positive, a loopful of specimen from the blood culture bottle was inoculated on Twin plate 32 (sheep blood agar + bromothymol blue agar; Kyokuto, Tokyo, Japan) at 37 °C under aerobic conditions for 1 day and on Twin plate 47 (sheep blood agar + chocolate agar; Kyokuto) at 37 °C under 5% CO_2_ for 1 day. If the anaerobic blood culture was positive, the specimen was inoculated onto Brucella agar (Kyokuto) at 37 °C under anaerobic conditions for 1 day. Biochemical tests were additionally performed using the Walkaway 96 Plus system (Beckman Coulter, Brea, CA, USA). Based on these results, bacteria were comprehensively identified. The susceptibility testing of antibiotics against identified bacteria was also performed using the Walkaway 96 Plus system (Beckman Coulter).

For catheter tip culture, catheter tips were placed in HK semi-solid medium (Kyokuto) at 37 °C for a maximum of 2 days to culture aerobic and anaerobic microorganisms. If bacteria had grown on the semi-solid medium, Gram staining was performed using the positive sample. Moreover, aliquots of culture were inoculated onto Twin 32 (Kyokuto), Twin 47 (Kyokuto), and Brucella agars (Kyokuto), similarly to the process for identification of positive blood culture samples. Other cultures from wounds, sputum, or drainage were also performed, if considered necessary by the physician.

### Extraction and purification of DNA

The tube containing the catheter was filled with purified water until the entire catheter was immersed. Water was passed through the catheter lumen by pipetting. After sonicating the specimen-containing tubes for 15 min in an ultrasonic bath (US-102; SND Co., Ltd., Suwa, Japan), 1 ml of each solution was transferred to clean 1.5-ml Eppendorf tubes. The solutions were concentrated to approximately 100 μl using a SpeedVac (Thermo Fisher Scientific, Waltham, MA, USA). Extraction and purification of DNA was performed using a NucleoSpin^®^ Soil (Macherey–Nagel, Düren, Germany) according to the manufacturer’s protocol.

### PCR amplification of 16S rDNA

The V3–V4 fragments of 16S rDNA were amplified from DNA extracts as described in the 16S Metagenomic Sequencing Library Preparation Protocol, Part # 15044223 Rev. B (Illumina Inc., San Diego, CA, USA) with the following modifications. PCR was performed in a total volume of 25 μl, containing 9.5 μl of DNA extract, 0.6 μM forward and reverse primers, and 12.5 μl of Tks Gflex™ DNA Polymerase Low DNA (TAKARA BIO INC., Otsu, Japan). PCR reaction conditions were as follows: initial denaturation for 1 min at 94 °C and 30 cycles consisting of 10 s at 98 °C, 15 s at 50 °C, and 15 s at 68 °C. After PCR amplification, 5 μl of reaction mixture was applied to gel electrophoresis using a 1.0% agarose gel and subsequently stained with 0.5 μg/ml of ethidium bromide for 30 min. The gel was analyzed using an ImageQuant LAS-4000 (GE Healthcare, Chalfont St. Giles, UK) under UV trans-illumination. The specimens that showed bands of the target size were regarded as positive. The amplified PCR products were purified using the Agencourt AMPure XP beads (Beckman Coulter) according to the manufacturer’s protocol.

### Index PCR

Index PCR was performed in a total volume of 25 μl, containing 2 μl of purified DNA sample, 2.5 μl each of index 1 and index 2 primers in the Nextera XT Index Kit (Illumina Inc.), 12.5 μl of Tks Gflex™ DNA Polymerase Low DNA (TAKARA BIO INC.), and 5.5 μl of pure water. PCR reaction conditions were as follows: initial denaturation for 1 min at 94 °C and 8 cycles consisting of 10 s at 98 °C, 15 s at 60 °C, and 15 s at 68 °C. PCR purification was performed as mentioned above. When two bands were observed after electrophoresis, the band of the desired size was purified using a NucleoSpin Gel and PCR Clean-up (Macherey–Nagel). The DNA was finally purified using the AMPure XP beads (Beckman Coulter) according to the manufacturer’s protocol. The concentration of DNA was measured using a Qubit 3.0 Fluorometer (Thermo Fisher Scientific) after staining with the Qubit dsDNA HS assay kit (Thermo Fisher Scientific) according to the manufacturer’s instructions.

### NGS and data analysis

The prepared libraries were subjected to sequencing of paired-end reads of 250 bp using the MiSeq Reagent Kit v3 on the MiSeq (Illumina) at the Biomedical Centre, Takara Bio. The number of reads per sample was between 843,574 and 1,329,426. Processing of the sequencing data, including operational taxonomic unit (OTU) definition and taxonomy assignment, was performed using CD-HIT-OTU 0.0.1 [25] and QIIME ver. 1.8 [26], respectively.

### Statistical analysis

Fisher’s exact and Mann–Whitney U tests were used for categorical and continuous data, respectively. *P* value < 0.05 indicated statistical significance in all analyses. Statistical analyses were performed using GraphPad Prism 6 (GraphPad Software, La Jolla, CA, USA).

## Results

### Patient and CVC characteristics

**Fig. 1:**
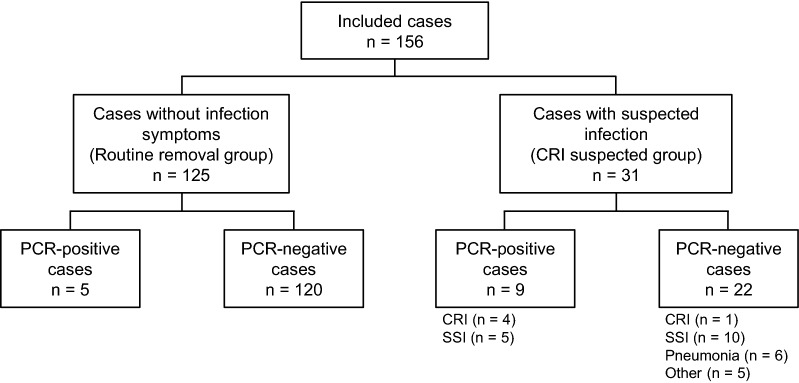
Flowchart of included cases

For the study, 156 patients (114 men; mean age 65.6 years) and their CVCs were enrolled (Fig. 1 and Table 1). In 141 patients (90.4%), catheterization was required for perioperative management. Underlying diseases are summarized in Table 1. In 125 patients (80.1%), catheters were removed because they were no longer needed, whereas in 31 patients (19.9%), catheters were removed due to the suspicion of CRI. There were significant differences in qSOFA score, body temperature, frequency of skin redness, frequency of antibiotic use post-removal, and the defervescence time after catheter removal between the two groups.

**Table 1:** Patient characteristics (n = 156)

	Total	Routine removal group	CRI-suspected group	*P* value
	(n = 156)	(n = 125)	(n = 31)	
Age, years (mean ± SD)	65.6 ± 10.8	65.3 ± 10.9	66.5 ± 10.3	0.645
Male, n (%)	114 (73.1%)	93 (74.4%)	21 (67.7%)	0.500
Underlying disease^a^				
Malignancy	126 (80.8%)	101 (80.8%)	25 (80.6%)	1.000
Hepatocellular carcinoma	49 (31.4%)	43 (34.4%)	6 (19.4%)	−
Pancreatic cancer	24 (15.4%)	20 (16.0%)	4 (12.9%)	−
Cholangiocarcinoma	11 (7.1%)	5 (4.0%)	6 (19.4%)	−
Other cancers	42 (26.9%)	33 (26.4%)	9 (29.0%)	−
Diabetes mellitus	40 (25.6%)	33 (26.4%)	7 (22.6%)	0.819
Chronic hepatitis/liver cirrhosis	21 (13.5%)	18 (14.4%)	3 (9.7%)	0.769
Chronic kidney disease	3 (1.9%)	2 (1.6%)	1 (3.2%)	0.488
Other diseases	30 (19.2%)	24 (19.2%)	6 (19.4%)	1.000
Immunosuppressive drug use (yes)	8 (5.1%)	6 (4.8%)	2 (6.5%)	0.659
qSOFA score point (mean and range)	0.2 (0–3)	0.1 (0–1)	0.5 (0–3)	< 0.001
Body temperature, °C (mean ± SD)	36.8 ± 0.8	36.6 ± 0.5	37.7 ± 1.1	< 0.001
Skin redness at the insertion site	5 (3.2%)	0 (0%)	5 (16.1%)	< 0.001
Insertion site of CVC				
Jugular	148 (94.9%)	120 (96.0%)	28 (90.3%)	0.196
Femoral	3 (1.9%)	2 (1.6%)	1 (3.2%)	0.488
Peripherally inserted CVC	5 (3.2%)	3 (2.4%)	2 (6.5%)	0.259
Number of lumen				
3	103 (66.0%)	84 (67.2%)	19 (61.3%)	0.533
2	48 (30.8%)	38 (30.4%)	10 (32.3%)	0.831
1	5 (3.2%)	3 (2.4%)	2 (6.5%)	0.259
Primary purpose for CVC insertion				
Perioperative	141 (90.4%)	114 (91.2%)	27 (87.1%)	0.500
Parenteral nutrition	9 (5.8%)	6 (4.8%)	3 (9.7%)	0.383
Intensive care	6 (3.8%)	5 (4.0%)	1 (3.2%)	1.000
Duration of catheterization, days (mean ± SD)	9.5 ± 9.0	9.0 ± 8.0	11.5 ± 12.2	0.600
Antibiotic use during the catheterization	153 (98.1%)	123 (98.4%)	30 (96.8%)	0.488
Antibiotic use after the removal	47 (30.1%)	22 (17.6%)	25 (80.6%)	< 0.001
Defervescence time from the catheter removal, day (mean ± SD)	0.8 ± 2.4	0.3 ± 0.6	3.4 ± 4.5	< 0.001

Data are presented as the number (percentage) of patients

^a^Some overlap exists

### Frequency of CRI and other infectious diseases based on the conventional method

Out of the 31 cases in the CRI-suspected group, five patients (16.1% and 3.2% of total) were definitively diagnosed with CRI, based on the positive result of conventional culture tests (Fig. 1 and Table 2). Moreover, surgical site infections (SSIs) were confirmed in 15 (48.4%), based on clinical, radiological, and laboratory findings, followed by pneumonia in six (19.4%).

**Table 2:** Comparison of PCR and conventional culture results in the CRI-suspected group (n = 31)

	Culture results		
	Positive	Negative	Total
PCR results			
Positive	4	6	10
Negative	1	20	21
Total	5	26	31

### PCR amplification of 16S rDNA from catheter specimens

**Fig. 2:**
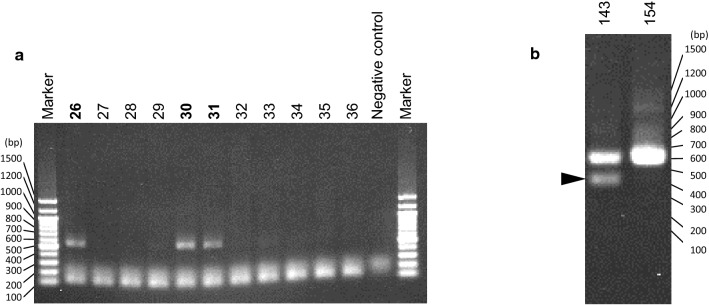
Representative agarose-gel images. **a** A representative agarose-gel image of PCR-amplified 16S rDNA. The number of specimens regarded as positive are shown in bold. PCR reaction of the negative control sample was performed without any DNA extracted from catheter specimen. The bands around 100 to 200 bp are considered to be primer dimers. **b** A representative agarose-gel image of index PCR products. The minor band, presumably due to nonspecific amplification, is indicated by an arrowhead

Representative results of agarose-gel electrophoresis are shown in Fig. 2. After index PCR, a minor band was observed below the main band in 50% of the samples, probably due to nonspecific amplification.

### Summary of cases with positive result from 16S rDNA-targeted NGS

**Fig. 3:**
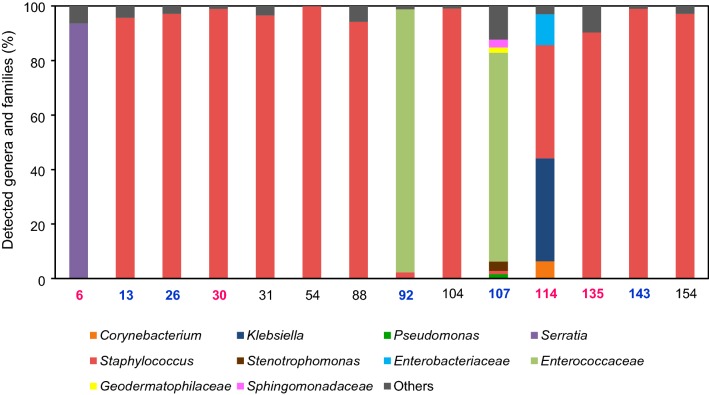
Bacterial compositions in catheter specimens. “Others” include families and genera that are less than 1%. The final diagnoses of patients from which magenta and blue specimens were derived were of CRI and SSI, respectively

Out of 156 CVC specimens, 14 (9.0%) showed positive results by the 16S rDNA-targeted NGS method. The details of clinical and bacteriological findings are shown in Table 3. Nine out of the above-mentioned 14 patients belonged to the CRI-suspected group, and five belonged to the routine removal group. Bacterial composition is shown in Fig. 3. The genus *Staphylococcus* was the most frequently detected. However, in specimen no. 114, only *Klebsiella pneumoniae* was identified by the culture test, whereas both *Klebsiella* (38.0% of all detected genera) and *Staphylococcus* (42.0%) were detected as the predominant bacteria.

**Table 3:** Clinical and bacteriological findings in cases with positive result from either NGS or conventional methods (blood and catheter tip culture)

No.	Age	Sex	BT	Skin redness	Bacteria detected by each method				Final diagnosis	Defervescence time (day)	Antibiotic treatment after the catheter removal	Outcome
					NGS (the genera or families account for more than 10% of the leads)	Blood culture	Catheter tip culture	Other cultures				
CRI suspected group												
NGS positive												
6	76	F	36.1	Yes	*Serratia*	NA	*Serratia marcescens*	NA	CRI	–	CMZ	Cure
13	59	M	37.6	No	*Staphylococcus*	NA	Negative	NA	SSI	2	CPFX	Cure
26	59	M	37.0	No	*Staphylococcus*	Negative	Negative	NA	SSI	1	FOM	Cure
30	76	M	40.0	Yes	*Staphylococcus*	*Staphylococcus epidermidis*	*Staphylococcus epidermidis*	NA	CRI	4	PIPC/TAZ	Cure
92	40	F	35.9	No	*Enterococcaceae*	Negative	Negative	NA	SSI	–	LVFX	Cure
107	74	F	37.0	No	*Enterococcaceae*	Negative	Negative	*Enterococcus faecalis* (drain culture)	SSI	1	IPM/CS	Cure
114	65	F	38.7	No	*Staphylococcus, Klebsiella*, *Enterobacteriaceae*	Negative	*Klebsiella pneumoniae*	NA	CRI	1	CEZ	Cure
135	61	M	39.0	No	*Staphylococcus*	*Staphylococcus aureus*	*Staphylococcus aureus*	NA	CRI	13	VCM	Death^a^
143	62	M	37.0	No	*Staphylococcus*	Negative	Negative	NA	SSI	1	LVFX	Cure
NGS negative												
112	62	M	37.5	Yes	Negative	*Staphylococcus epidermidis*	Negative	NA	CRI	1	VCM	Cure
Routine removal group												
NGS positive												
31	80	F	36.4	No	*Staphylococcus*	NA^b^	NA	NA	Post infection (DNAemia)	–	No	–
54	70	M	36.3	No	*Staphylococcus*	NA	NA	NA	Contamination	–	No	–
88	65	F	36.6	No	*Staphylococcus*	NA	NA	NA	Contamination	–	No	–
104	82	F	35.9	No	*Staphylococcus*	NA	NA	NA	Contamination	–	No	–
154	52	M	36.8	No	*Staphylococcus*	NA	NA	NA	Contamination	–	No	–

*BT* body temperature, *NA* not applicable, *CEZ* cefazolin, *CMZ* cefmetazole, *CPFX* ciprofloxacin, *FOM* fosfomycin, *IPM/CS* imipenem/cilastatin, *LVFX* levofloxacin, *PIPC/TAZ* piperacillin/tazobactam, *VCM* vancomycin

^a^Although the symptom temporarily improved after catheter removal, CRI finally led to a fatal outcome. An autopsy examination revealed *S. aureus* bacteraemia

^b^*Staphylococcus capitis* was identified 9 days before catheter removal. The catheter was, nevertheless, inserted due to therapeutic requirements

### Comparison of PCR and conventional culture results in the CRI-suspected group

Among the 31 cases in the CRI-suspected group, comparison between the NGS results and conventional culture results for the diagnosis of CRI (blood culture and catheter tip culture) is shown in Table 2. A high level of agreement existed between the two methodologies, with sensitivity, specificity, and accuracy values of 80.0, 76.9, and 77.4%, respectively. In the four patients having confirmed CRI, with both 16S rDNA-targeted NGS and culture results positive, pathogens identified by conventional cultures were all detected by the 16S rDNA-targeted NGS method (Table 3).

### Discrepant case with culture-positive but NGS-negative outcome in the CRI-suspected group

In one patient with confirmed CRI (no. 112), although *Staphylococcus epidermidis* was identified using the conventional culture method, the NGS method did not detect the pathogen (Table 3).

### Discrepant case with NGS-positive but culture-negative outcome in the CRI-suspected group

Five discrepant cases (nos. 13, 26, 92, 107, and 143) with NGS-positive but culture-negative outcomes were obtained; all were diagnosed as SSI. Moreover, in one of the five cases (no. 107), *Enterococcaceae* detected by the NGS method were also identified as *Enterococcus faecalis* in abdominal abscess drainage cultures, although neither blood nor catheter tip cultures were positive.

### NGS-positive cases in the routine removal group

Of the 125 patients in the routine removal group, the 16S rDNA-based NGS method showed a false-positive result in five (4.0%); the genus *Staphylococcus* was detected in all (Table 3). There was no clinical evidence of infection in four patients, while *S. capitis* bacteremia had occurred just 9 days before catheter removal in one case (no. 31).

## Discussion

In the present study, we have developed a new molecular diagnostic method using targeted NGS of bacterial 16S rDNA extracted from CVC surfaces. To the best of our knowledge, this is the first report demonstrating that NGS can detect causative bacteria in samples obtained from catheter surfaces from patients with CRI. This novel method showed comparable sensitivity with conventional culture methods in the CRI-suspected group and a low false-positive rate in the routine removal group. Hence, we believe that using targeted NGS of bacterial 16S rDNA to detect causative pathogens in catheter tip samples is a clinically promising method.

*Staphylococcus* was the most frequent causative pathogen of CRI in the present study, consistent with previous reports [13, 14, 27, 28]. In four out of five cases with definitive CRI, there was agreement regarding the causative pathogens between the conventional culture and the 16S rDNA-based NGS method at the genus level. Meanwhile, in the one remaining case with definite CRI (no. 112), *S. epidermidis* was identified by only blood culture, not by the NGS method nor catheter tip culture. It was presumed that both NGS and catheter tip culture were negative because no or a very small amount of bacteria below the detection limit were attached to the segments of catheters used for the assays. Taken together, in cases where a certain amount of bacterial cells are attached to the CVC specimens tested, the NGS method may be useful as an auxiliary or alternative diagnostic tool for CRI to overcome the limitations of conventional culture tests, such as the low sensitivity of the tests under the influence of antibiotics.

Similar to previous reports [29–31], 83.9% of patients in the suspected group were proven to not have CRI after assessment of catheter tip and blood cultures in the present study. Among the samples, SSI was the most frequent diagnosis in 15 cases. In one such case (no. 107), where *E. faecalis* was identified by the abscess drainage culture, *Enterococcaceae* were also detected in the catheter tip sample based on the NGS method; however, blood and catheter tip culture were both negative. This result suggested that the NGS method has higher sensitivity for detecting an early bloodstream infection than blood culture test.

One advantage of NGS is that one sequence run in a single protocol enables identification of polymicrobial pathogens [16]. Similar to previous studies that revealed the utility of NGS for detecting multiple causative agents in infectious diseases [19, 22, 23], in the present study, multiple causative pathogens were simultaneously detected in CVC specimens by NGS in two cases (nos. 107 and 114) (Fig. 3); meanwhile, the culture method identified only one predominant bacterium detected by NGS (Table 3). This difference might be due to culture conditions. Specifically, the bacteria that can grow under the tested culture conditions are primarily identified, whereas other bacteria for which the culture conditions are not suitable may not be identified. If antibiotic therapy for the bacterial infection identified by culture is ineffective for other undetected bacteria, infection with the latter can worsen, leading to treatment failure. Therefore, the NGS method, which can detect diverse causative bacteria simultaneously, is more useful in selecting appropriate antibiotics, compared to the conventional culture methods.

Among the five NGS-positive cases in the routine removal group, four (nos. 54, 88, 104, and 154) did not show any infectious symptom before and after removal of the catheter, yet *Staphylococcus* was detected. This may be explained by contamination or physiological translocation of bacteria from skin or the nasal cavity, which did not induce bloodstream infection. In the remaining case (no. 31), although *Staphylococcus* was detected, *S. capitis* bacteremia had developed 9 days before the removal. This positive result was surmised as DNAemia. Overall, in the routine removal group, the false-positive rate of the NGS method was as low as 4% (5/125 cases). We believe that the NGS method rarely shows false-positives and thus is a reliable test for the accurate detection of the causative pathogens.

Currently, NGS requires a relatively long turnaround time and high expense [32]. Indeed, our NGS method takes several days for bacterial identification at the genus level, although the method can simultaneously process up to 384 specimens. Combination of our method with a recently reported new technique, which can determine bacterial composition at the species level by sequencing nearly full-length 16S rDNA using a nanopore sequencer within 2 h [33], may enable more rapid and high-resolution identification of causative bacteria from catheter tip samples. In addition, although the cost of NGS is declining, it is still expensive to use for routine diagnosis in clinical settings. To reduce running costs, it would be desirable to examine a large number of samples at the same time. Further cost reduction due to broader application of NGS in clinics is expected.

The present study has some limitations. First, the bacterial 16S rDNA-targeted NGS method is not able to detect fungi such as *Candida*, which is one of the primary causative pathogens of CRI in patients with malignancies [4]. However, in theory, the NGS method can also detect fungi from amplicon libraries composed of internal transcribed spacer (ITS) 1 and 2 lesions, which fungi possess, as previously reported [34]. Second, as the definition and methodology for CRI are slightly different from those recommended by the international guideline for practice [35], the interpretation of our preliminary clinical data might be limited. Nonetheless, the differences did not hinder evaluation of the applicability of the NGS method in this study. Third, in the routine removal group, we did not perform the conventional culture test. Therefore, we cannot distinguish between contamination by NGS or by true colonization. To solve these problems and to confirm the validity of targeted NGS of bacterial 16S rDNA and fungal ITS lesions using catheter tip samples in comparison to conventional methods, further clinical studies that follow the guidelines and include culture methods for the routine removal group are required.

## Conclusions

In conclusion, this preliminary clinical study demonstrated that the 16S rDNA-targeted NGS method correctly detected CRI causative pathogens in catheter tip samples. We believe that 16S rDNA-targeted NGS of catheter tip samples is a promising diagnostic tool in patients with CRI. Moreover, since the principle of this molecular diagnostic method can also be applied to detect pathogens on other devices, such as pacemakers, vascular grafts, and orthopedic implants, targeted NGS of bacterial 16S rDNA can contribute to the diagnosis of other device-related infections.

## Abbreviations

CRI: catheter-related infection
CVC: central venous catheter
ITS: internal transcribed spacer
NGS: next-generation sequencing
OTU: operational taxonomic unit
PCR: polymerase chain reaction
qSOFA: quick sequential (sepsis-related) organ failure assessment
SSI: surgical site infection
